# Metagenomics Reveals Sex-Based Differences in Murine Fecal Microbiota Profiles Induced by Chronic Alcohol Consumption

**DOI:** 10.3390/ijms252312534

**Published:** 2024-11-22

**Authors:** Manuel Domínguez-Pino, Susana Mellado, Carlos M. Cuesta, Rubén Grillo-Risco, Francisco García-García, María Pascual

**Affiliations:** 1 Computational Biomedicine Laboratory, Príncipe Felipe Research Center, C/Eduardo Primo Yúfera, 3, 46012 Valencia, Spain; manolouvus@gmail.com (M.D.-P.); rgrillo@cipf.es (R.G.-R.); fgarcia@cipf.es (F.G.-G.); 2 Department of Physiology, School of Medicine and Dentistry, University of Valencia, Avda. Blasco Ibáñez, 15, 46010 Valencia, Spain; susana.mellado@uv.es (S.M.); carlosmcd90@gmail.com (C.M.C.)

**Keywords:** fecal microbiota, ethanol, sex differences, TLR4, 16S rRNA, inflammation

## Abstract

Chronic ethanol exposure induces an inflammatory response within the intestinal tract, compromising mucosal and epithelial integrity and leading to dysbiosis of the gut microbiome. However, the specific roles of the gut microbiota in mediating ethanol-induced effects, as well as their interactions with the immune system, remain poorly characterized. This study aimed to evaluate sex-based differences in fecal microbiota profiles induced by chronic alcohol consumption and to assess whether TLR4 is involved in these effects. We analyzed the 16S rRNA gene sequencing of fecal samples from male and female wild-type (WT) and TLR4-knockout (TLR4-KO) mice with and without chronic ethanol exposure over a three-month period. Our findings provide evidence, for the first time, that male mice are more susceptible to the effects of ethanol on the fecal microbiota, since ethanol exposure induced greater alterations in the Gram-negative and -positive bacteria with immunogenic capacity in the WT male mice than in the female mice. We also demonstrate that the absence of immune receptor TLR4 leads to different microbiota in both sexes, showing anti-inflammatory and protective properties for intestinal barrier function and resulting in a phenotype more resistant to ethanol’s effects. These findings may open new avenues for understanding the relationship between gut microbiota profiles and inflammation in the digestive system induced by chronic alcohol consumption.

## 1. Introduction

The intestinal microbiota comprise tens of trillions of microorganisms, encompassing over 1000 identified bacterial species, the vast majority of which belong to the phyla *Firmicutes* (Gram-positive) and *Bacteroidetes* (Gram-negative) [1]. The gastrointestinal tract and the intestinal microbiota have a symbiotic relationship that supports physiological functions. However, the disruption of intestinal microbiota homeostasis, known as dysbiosis, has been linked to pathological conditions affecting the entire body, including gut inflammatory processes [1] and behavioral impairments [2,3,4]. Various factors, such as alcoholic beverage consumption, may contribute to the development and progression of many diseases by altering the gut microbiota. In fact, several studies have demonstrated the effects of alcohol on the gut microbiota in both rodents [1,5,6] and humans [1,5,7]. These changes are thought to be relevant for alcohol-associated pathologies, as interventions that modify the intestinal microbiota have been shown to reduce certain alcohol-related diseases [1].

Our previous studies have demonstrated that ethanol activates toll-like receptor 4 (TLR4), initiating an innate immune response that promotes the release of cytokines and other inflammatory mediators [8,9]. Furthermore, it has been shown that the effects of ethanol on inflammation in the brain and plasma are sex-dependent, with females exhibiting greater vulnerability than males to the toxic effects of alcohol [10]. Although several animal and human studies have reported that sex as a key factor influencing the gut microbiota, this association has not yet been thoroughly investigated.

Similar to ethanol, the structural components of Gram-negative bacteria, such as lipopolysaccharide (LPS), can activate TLR4 or other TLRs, triggering gut inflammatory processes [11]. Overstimulation of the innate immune system that is driven by gut dysbiosis and/or small intestinal bacterial overgrowth, coupled with increased intestinal barrier permeability, can result in both localized and systemic inflammation and enteric neuroglial activation, as seen in certain neurodegenerative diseases like Parkinson’s disease [12]. Conversely, some evidence suggests that certain bacterial strains can prevent inflammation and promote mucosal repair and recovery in a healthy intestine [11]. However, the influence of TLR signaling on commensal–host interactions appears to be context-dependent. In this sense, a deeper understanding of the relationship between the gut microbiota and TLRs’ immune responses could pave the way for novel therapeutic strategies focused on modulating the gut microbiota composition, restoring balance to the innate immune response, and enhancing intestinal epithelial barrier function in pathological conditions.

Considering that chronic ethanol consumption can modify the gut microbiota and that sex differences have been observed in ethanol’s effects, the present study analyzed, through a metagenomic approach, whether chronic ethanol treatment differentially modified the fecal microbiota in female and male mice, and it also analyzed the involvement of TLR4 in these effects. Our results demonstrated that the fecal microbiota profile of ethanol-treated WT male mice exhibited higher abundances of Gram-negative and -positive bacteria with greater immunogenic capacity compared to the WT females. Additionally, the deficiency of the TLR4 receptor altered the microbiota composition in the ethanol-treated TLR4-KO mice of both sexes, leading to greater abundances of bacterial species linked to anti-inflammatory effects and intestinal barrier protection, promoting a phenotype more resistant to the effects of ethanol. This study analyzed, for the first time, the involvement of sex differences and the role of TLR4 in the alterations to the fecal microbiota induced by chronic alcohol consumption.

## 2. Results

### 2.1. Effect of Ethanol Consumption on Fecal Bacterial Diversity in Male and Female Mice

**Figure 1:**
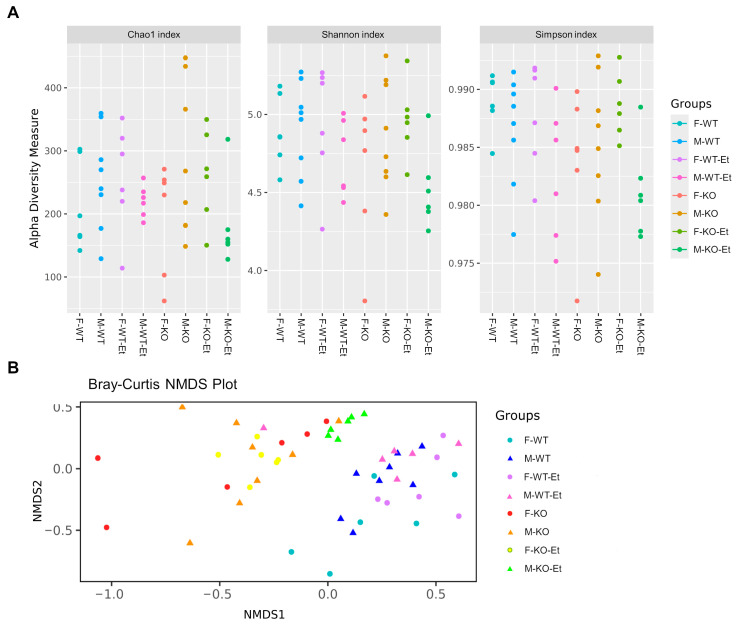
Alpha and beta diversity in the fecal microbiota of the WT and TLR4-KO mice with chronic alcohol exposure. (**A**) Alpha diversity was measured to assess the microbial richness and evenness within each sample group using the Chao1, Shannon, and Simpson indices. The Chao1 index estimated species richness by including rare species. The Shannon index measured both richness and evenness, providing valuable insight into species uniformity across our samples. The Simpson index complemented the Shannon by emphasizing the dominant species, allowing us to observe whether specific taxa were disproportionately abundant due to treatment or genotype differences. (**B**) A beta diversity plot based on Bray–Curtis distances using non-metric multidimensional scaling (NMDS) was used to visualize the differences in the microbiota community compositions among the experimental groups based on Bray–Curtis dissimilarities, associated with both the presence/absence and abundance of taxa. Each point represents a sample’s microbial profile, and the clustering indicates similarities in the microbial communities between the samples. The PERMANOVA (F-value: 3.0107, R-squared: 0.3238, *p*-value: 0.00099) was used to statistically assess the group clustering.

The bioinformatic strategy used in this metagenomics study yielded an average of 24,815 sequences per sample, with a standard deviation of 16,805 (Table S1). First, we analyzed the alpha diversity, or species richness, in murine feces by examining various indices (Figure 1A). Specifically, the alpha diversity not only measured the distribution of bacterial species within a sample but also assessed how evenly distributed these organisms were in terms of abundance. The Chao1 index estimated the total number of species in a community, including rare species that may not have been observed. This index indicated that, while the control TLR4-KO male mice showed higher alpha diversity, the control TLR4-KO female mice had lower diversity compared to the other experimental groups. Furthermore, as shown in Figure 1A, we also analyzed species diversity through the Shannon and Simpson indices, which measure species abundance and uniformity and the probability that two randomly selected bacteria belong to different species, respectively. These results showed no significant differences between the fecal samples from the male and female mice, between the control and ethanol-treated mice, or between the WT and TLR4-KO mice.

Next, a beta diversity analysis of the fecal microbiota was conducted to evaluate structural similarities in the microbiota communities across the individual samples from all the experimental groups using a Bray–Curtis-based non-metric multidimensional scaling (NMDS) plot (Figure 1B). Our results demonstrated a separation between the fecal samples from the WT and TLR4-KO mice. However, no dispersion was observed between the males vs. females or the control vs. ethanol-treated mice. Thus, the samples from the WT and TLR4-KO mice showed distinct clustering patterns, indicating compositional shifts based on genotype, while the differences due to sex and ethanol treatment were less pronounced.

### 2.2. Effect of Ethanol Consumption on the Relative Abundance of Fecal Bacteria in Male and Female Mice

**Figure 2:**
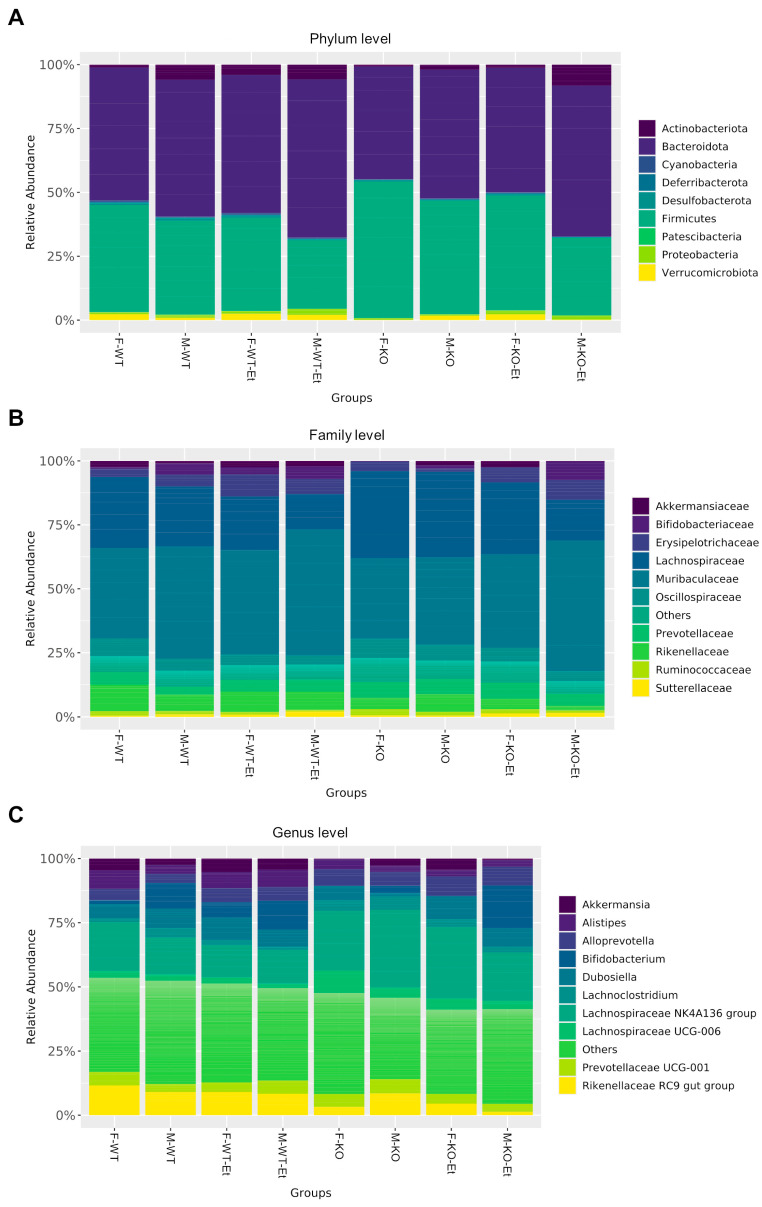
The relative species abundance in the fecal samples at the (**A**) phylum level, (**B**) family level, and (**C**) genus level.

**Figure 3:**
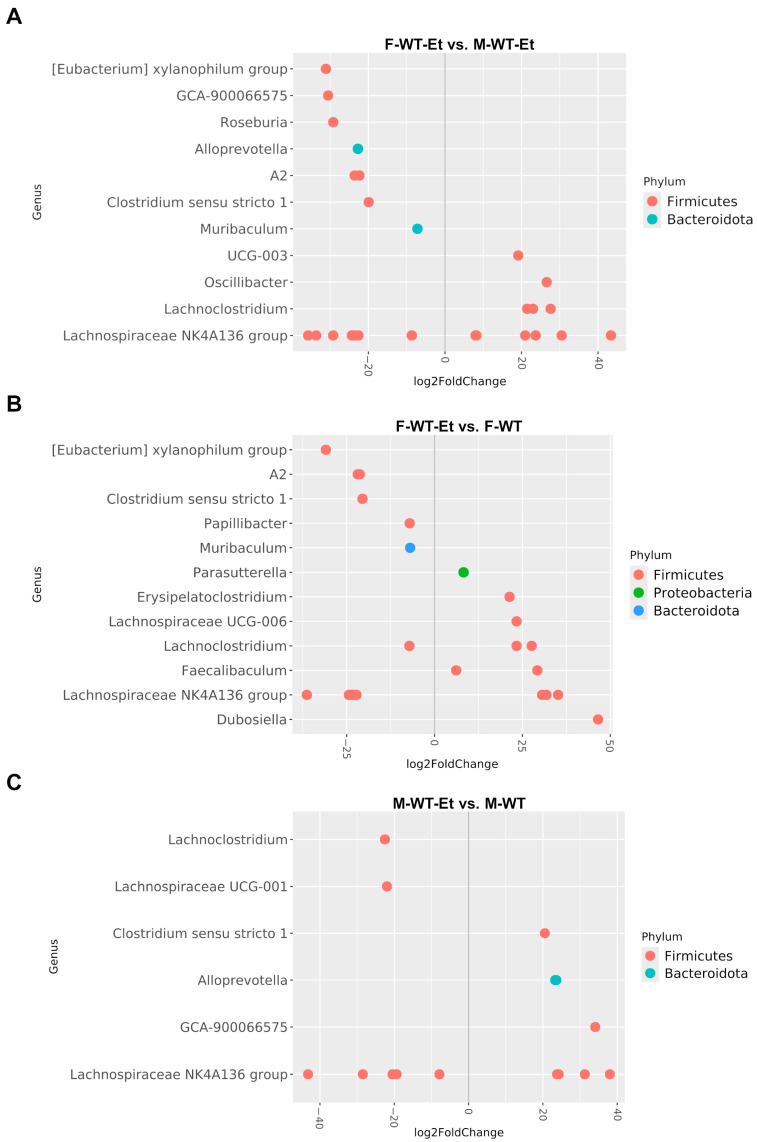
Summary of the differential abundance analysis at the genus level. (**A**) Results of the comparison between the ethanol-treated WT male and female mice (F-WT-Et vs. M-WT-Et). (**B**) Results of the comparison between the ethanol-treated and untreated WT female mice (F-WT-Et vs. F-WT). (**C**) Results of the comparison between the ethanol-treated and untreated WT male mice (M-WT-Et vs. M-WT). The positive log2 fold changes indicated an over-representation of the taxa in the first group compared to the second, while the negative log2 fold changes indicated an under-representation of the taxa in the first group compared to the second. The dot colors represent the phylum corresponding to each genus. Only the ASVs with an adjusted *p*-value of <0.05 are shown.

Although our analysis revealed that alpha diversity was not significantly influenced by ethanol consumption or sex, we further examined the relative abundance of microbiota at the phylum, family, and genus levels (Figure 2). It has been described that the gut microbiota exhibit individual-specific compositions at the genus and species levels; however, they show greater conservation at the phylum level, where *Bacteroidetes* and *Firmicutes* are most prominent, followed by *Proteobacteria* and *Actinobacteria* [13]. In line with this, Figure 2A shows the relative abundance of bacteria in feces. Two phyla, *Bacteroidota* and *Firmicutes*, predominate, while *Actinobacteriota* and *Verrucomicrobiota* are represented to a lesser extent in some groups. At the family level (Figure 2B), we observed greater diversity between the different experimental groups, making it challenging to establish similarity patterns among the groups. Differences, although not significant, were observed based on sex, ethanol treatment, and genotype. As in the family taxonomic category, reducing the taxonomy to the genus level revealed more pronounced and variable differences among the groups compared to the family and phylum levels. Thus, the differential abundance of fecal microbiota at the genus level was further analyzed, as shown in Figure 3.

### 2.3. Sex Differences in Bacterial Differential Abundance Induced by Chronic Alcohol Consumption

Bacteria abundance alterations were evaluated using the DESeq2 package to identify amplicon sequence variants (ASVs), which provide a higher-resolution and more sensitive alternative to using the traditional OTU table [14], and the results showed statistically significant differences in abundance (adjusted *p*-value of <0.05) across the comparisons in our study. Table 1 shows the analysis of the differentially abundant taxa for each comparison. An under- or over-represented value (negative or positive log2 fold change) indicated significant differences for the variable in the first group of each comparison. The “Non-differential represented” variable referred to bacterial species that did not exceed the significance threshold. Considering the variables of sex, ethanol treatment, and genotype, the comparisons revealed significant differences in both high- and low-abundance bacteria (Table 1).

#### 2.3.1. Bacterial Differential Abundance in the WT Female and Male Mice

Figure 3 shows the bacterial differential abundance between the WT mice with and without ethanol treatment, highlighting the sex differences in the fecal microbiota compositions. First, we compared the ethanol-treated WT mice from both sexes (Figure 3A). Our results demonstrated that alcohol consumption in the WT male mice induced an enrichment of the Gram-negative bacteria genera *Alloprevotella* and *Muribaculum* (phylum *Bacteriodota*), as well as in Gram-positive bacteria, such as *Roseburia* and the *Eubacterium xylanophilum* group (phylum *Firmicutes*), compared to the WT female mice. Conversely, other Gram-positive bacteria, such as *Lachnoclostridium* and *Oscillibacter*, were more abundant in the ethanol-treated WT female mice than in the WT male mice. Regarding the comparison between the untreated and ethanol-treated WT mice (Figure 3B,C), 12 specific ASVs were statistically affected in the females, whereas the males showed only 6 specific ASVs. Figure 3B shows a higher abundance of *Parasutterella* (phylum *Proteobacteria*) and a lower abundance of *Papillibacter* (phylum *Firmicutes*) in response to ethanol treatment in the ethanol-treated WT female mice compared to the control mice. Table 2 summarizes only the phyla with the most significant adjusted *p*-values for each comparison.

#### 2.3.2. Bacterial Differential Abundance in the TLR4-KO Female and Male Mice

**Figure 4:**
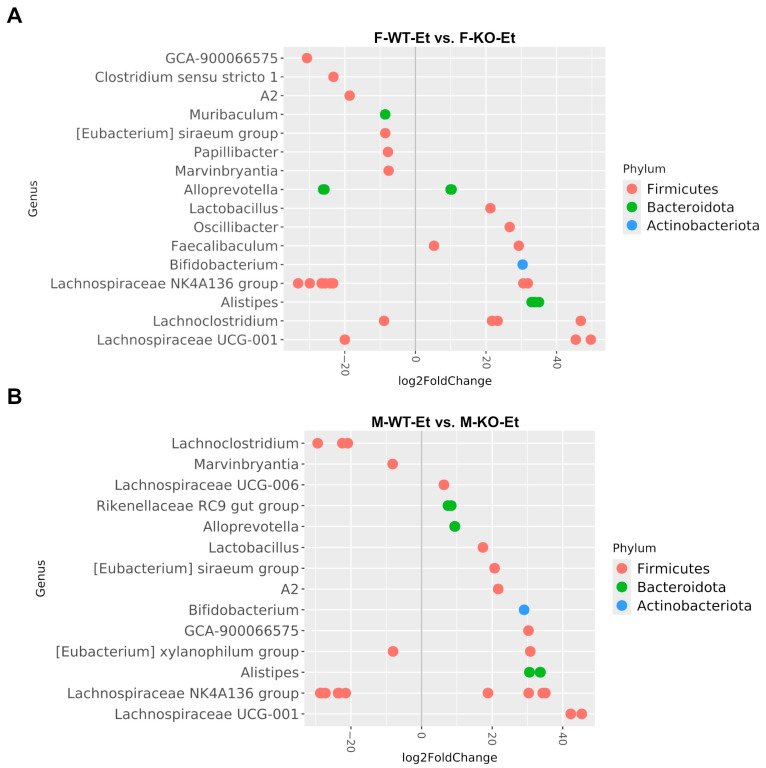
Summary of the differential abundance analysis at the genus level. (**A**) Results of the comparison between the ethanol-treated WT and TLR4-KO female mice (F-WT-Et vs. F-KO-Et). (**B**) Results of the comparison between the ethanol-treated WT and TLR4-KO male mice (M-WT-Et vs. M-KO-Et). The positive log2 fold changes indicated an over-representation of the taxa in the first group compared to the second, while the negative log2 fold changes indicated an under-representation of the taxa in the first group compared to the second. The dot colors represent the phylum corresponding to each genus. Only the ASVs with an adjusted *p*-value of <0.05 are shown.

Next, we evaluated the differential abundance of the bacterial species in both sexes between the ethanol-treated WT and the TLR4-KO mice (Figure 4). Our results showed an enrichment of *Alistipes* and *Alloprevotella* (Gram-negative bacteria) in both sexes of the ethanol-treated WT mice compared to the TLR4-KO mice. Regarding the Gram-positive bacteria, *Bifidobacterium* and *Lactobacillus* were more abundant in the ethanol-treated WT mice compared to the ethanol-treated TLR4-KO mice, while *Marvinbryantia* showed a lower fecal abundance in the ethanol-treated WT mice than in the TLR4-KO mice. Table 3 summarizes only the phyla with the most significant adjusted *p*-values for each comparison.

## 3. Discussion

Alcohol consumption exerts significant effects on the gut microbiome, contributing to dysbiosis and heightened systemic inflammation [1]. We previously demonstrated that chronic ethanol exposure altered fecal microbiota and upregulated several inflammatory, structural, and permeability genes in the colon of WT male mice, whereas no changes were observed in ethanol-treated TLR4-KO mice. In addition, a negative correlation between bacteria species and structural genes was found in ethanol-treated animals [6]. Our evidence has also indicated that ethanol can induce sex-specific differences in the inflammatory immune response in both humans and mice [10,15,16]. In this context, the aim of this study was to analyze whether ethanol differentially modifies the fecal microbiota in female and male mice, and whether TLR4 also participates in these changes. Interestingly, the microbiota profiles of the ethanol-treated WT male mice showed higher abundances of both Gram-negative and -positive bacteria (e.g., *Alloprevotella*, *Muribaculum*, *Roseburia*, the *Eubacterium xylanophilum* group, *Lachnoclostridium,* and *Oscillibacter*), with higher immunogenic capacity compared to the WT females. Additionally, the deficiency in the TLR4 receptor altered the microbiota composition in the ethanol-treated TLR4-KO mice of both sexes (e.g., *Alistipes*, *Alloprevotella*, *Marvinbryantia*, *Bifidobacterium,* and *Lactobacillus*), leading to a higher enrichment of bacterial species associated with anti-inflammatory properties and intestinal barrier protection and promoting a phenotype more resistant to the effects of ethanol.

Chronic alcohol consumption causes dysbiosis, which is associated with an increase in the growth of Gram-negative bacteria [1]. This enrichment in Gram-negative bacteria is linked to intestinal inflammation and pathologies related to dysbiosis due to the presence of the endotoxin LPS in their outer membranes [17]. In this context, our results demonstrated that alcohol consumption in the WT male mice induced an enrichment in the genus of Gram-negative bacteria, *Alloprevotella* and *Muribaculum* (phylum *Bacteriodota*), compared to the WT female mice. The genus *Alloprevotella* exhibited increased inflammatory properties, and it has been considered clinically important as a pathobiont involved in promoting chronic inflammation [18]. A recent study reported that the metabolites of *Muribaculum* can induce an exacerbated production of pro-inflammatory cytokines [19]. Other Gram-positive bacteria, such as *Roseburia* and the *Eubacterium xylanophilum* group, have been strongly correlated with pro-inflammatory cytokine levels in the gut microbiota of a temporal lobe epilepsy rat model [20]. Both bacteria showed higher abundances in the WT male mice than in the female mice after alcohol consumption. Additionally, an enrichment of *Parasutterella* (phylum *Proteobacteria*) in response to ethanol treatment was observed in the WT female mice. Although *Parasutterella* is a Gram-negative bacterium that is part of the healthy fecal core microbiome in both humans and mice gastrointestinal tracts [21], its marked expression has been associated with chronic intestinal inflammation, suggesting its involvement in the onset and development of irritable bowel syndrome [22]. It is also important to note that chronic ethanol consumption can alter bile acid metabolism in various body compartments [23], which is linked to disease development and dysbiosis [24].

Intestinal inflammation in humans is linked to dysbiosis or an imbalance in the microbiota, which is marked by a loss of microbial diversity, a decline in beneficial bacteria, and/or an expansion of pathogenic bacteria. Studies have shown that an abundance of potentially pathogenic bacteria, such as *Enterobacteriaceae* and *Streptococcaceae*, along a reduction in beneficial taxa like *Lachnospiraceae* may influence prognoses in patients with cirrhosis [25]. A reduction in *Lachnoclostridium* and *Oscillibacter*, which have anti-inflammatory potential [26,27], has been observed in ethanol-treated WT male mice. *Oscillibacter,* in particular, has been demonstrated to generate anti-inflammatory molecules (e.g., IL-6, TNF-α, and IL-2) and suppress Th17 polarization, thus promoting the development of type 1 regulatory T cells with anti-inflammatory functions within the intestine [28]. Other anti-inflammatory compounds, such as butyric acid, are produced by various bacteria (e.g., the *Papillibacter* species) in the gastrointestinal tract, supporting intestinal health and stability [29]. Butyric acid and butyrate are well known for their benefits to intestinal barrier integrity and mucosal immunity [30]. Patients with chronic alcohol overconsumption have shown lower concentrations and percentages of butyric acid, which may contribute to a pro-inflammatory direction [31]. In our study, a decrease in the abundance of *Papillibacter* was also observed in the ethanol-treated WT female mice. Therefore, chronic alcohol consumption promotes sex differences in bacterial abundance related to immunogenic capacity and healthy functions, suggesting that these bacterial alterations induced by ethanol were more pronounced in the WT male mice than in the females. Several studies have reported sex-based variations in gut microbiota composition. In a murine model of colitis, males displayed more pronounced colonic inflammation compared to females [32]. Furthermore, probiotic administration to rats exposed to water avoidance stress significantly reduced inflammatory cytokine levels only in female rats, indicating a sex-dependent difference in colonic microinflammation [33].

Changes in gut microbiota and gut permeability are linked to depression, anxiety, and cravings in alcohol use disorder (AUD), potentially reinforcing negative drinking behaviors [34]. Moreover, dysbiosis has been linked to depression, contributing to a heightened tendency to consume alcohol within the AUD population [35], which may exacerbate the severity of the disorder [36]. In this context, our results suggested that the male mice may have been at greater risk of experiencing the negative consequences of addictive-like behavior compared to the female mice due to the alcohol-induced alterations in bacterial abundance. In humans, drug use prevalence and the likelihood of developing dependence or abuse are generally higher in males; however, women tend to progress more rapidly from initial use to dependence [37]. Although the impacts of intestinal bacteria on neurobiological processes in individuals with substance use disorders remain largely unexplored [36], important sex-related differences exist that should be taken into account when optimizing treatments [37]. For example, using probiotics or prebiotics to modulate the gut microbiota offers a promising and safe therapeutic strategy for managing alcohol dependence [36].

A fundamental distinction exists in the regulation and function of TLRs within healthy versus inflamed intestinal mucosa, representing a delicate balance between host protection and tissue damage. In a healthy host, basal TLR signaling is essential for protective immune responses and tissue repair, both of which are vital for maintaining mucosal and commensal homeostasis [11]. Conversely, alcohol abuse induces a TLR4-dependent inflammatory response, leading to dysbiosis and gut barrier disruption [6,38]. The enrichment of pathogenic Gram-negative bacteria potentiates inflammatory liver damage in patients with chronic excessive use of alcohol [38]. Our results revealed an increase in *Alistipes* and *Alloprevotella* (Gram-negative bacteria) in both sexes of the WT mice treated with ethanol compared to the TLR4-KO mice. These species thrive in an inflamed environment, promoting inflammation and tumor formation [18,39]. Conversely, *Marvinbryantia* showed a lower fecal abundance in the ethanol-treated WT mice compared to the TLR4-KO mice. This bacterium has been negatively correlated with the serum levels of IL-6, TNF-α, and IL-1β, contributing to various cardioprotective effects [40]. Although certain bacteria, such as *Bifidobacterium* and *Lactobacillus*, might influence healthy functions [41], our results revealed an increase in these species in the ethanol-treated WT mice compared to the ethanol-treated TLR4-KO mice for both sexes. However, both species decompose alcohol into the carcinogenic and toxic compound acetaldehyde. At high concentrations, this compound has been shown to disrupt microbiota balance and directly injure hepatocytes [34]. Indeed, a past study on patients with alcohol dependence syndrome revealed an increased abundance of *Bifidobacterium* [42].

In this study, there were some limitations. First, the findings related to the gut microbial community in our experimental model were limited to taxonomic features at the genus levels, which are typically used when working with short-length 16S rRNA sequences [43]. Accurate identification at the species level through metataxonomics requires full-length 16S rRNA sequences [44], which future studies should incorporate to better elucidate the impacts of alcohol consumption on mouse fecal microbiota. A second limitation of this study is the small sample size used, which affected the variability within the same experimental group. Additionally, while some studies have shown the effects of sex differences on the gut microbiota, the magnitude of this contribution remains unclear compared to other factors such as medication and diet [45].

## 4. Materials and Methods

### 4.1. Animal Model and Alcohol Treatment

Male and female C57BL/6J WT (Harlan Ibérica S.L., Barcelona, Spain) and TLR4 knockout (TLR4-KO) mice were used. The TLR4-KO mice were kindly provided by Dr. S. Akira (Osaka University, Osaka, Japan) with C57BL/6J genetic backgrounds. The animals were kept under controlled light/dark conditions (12/12 h) at a temperature of 23 °C in 60% humidity. The animal experiments were carried out in accordance with the guidelines approved by the European Communities Council Directive (86/609/ECC) and Spanish Royal Decree 53/2013 and with the approval of the Ethical Committee of Animal Experimentation of the Príncipe Felipe Research Center (Valencia, Spain) and the Generalitat Valenciana on 18 December 2015 (Project identification code: 201 SNSC/PEA/00247).

**Figure 5:**
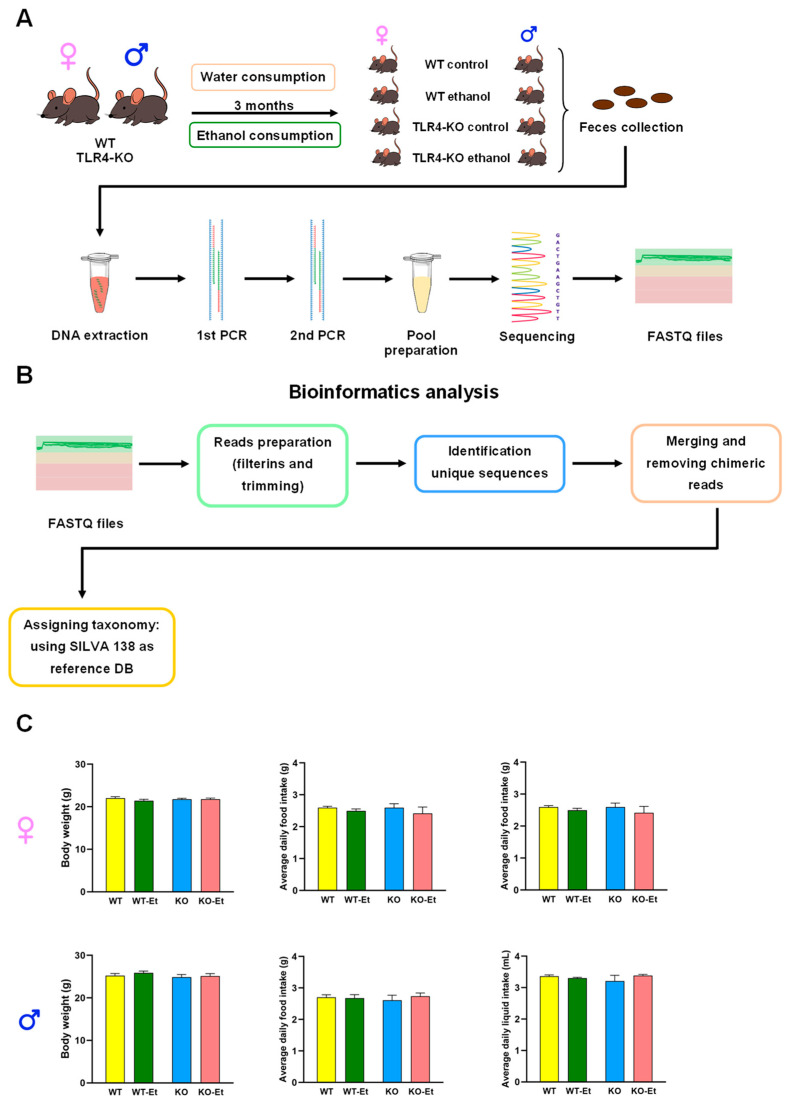
Schematic workflow of the experimental design and analysis. (**A**) Female and male C57BL/6J mice, with and without TLR4 deletion, were either administered water containing 10% (*v*/*v*) ethanol at the 2 month time point or maintained as parallel untreated controls. Fecal samples were collected from all mice at 5 months (3 months of ethanol treatment). DNA was extracted from the feces, and genomic 16S rRNA was sequenced. (**B**) The 16S rRNA gene sequencing analysis. (**C**) Bar graphs representing the body weights of all animals at the end of the ethanol treatment, along with the average daily food and liquid intake over the 3 months of ethanol treatment. The data represent means ± SEMs, with n = 6–8 mice/group.

The fifty-two two-month-old animals used were housed (two–four animals/cage) and divided into eight groups (six–eight mice/group), with WT and TLR4-KO mice for the control conditions for both sexes and WT and TLR4-KO mice for the chronic alcohol treatment for both sexes. The mice were treated with water (WT and KO) or water containing 10% (*v*/*v*) alcohol (WT-Et and KO-Et) for 3 months. They had ad libitum access to a solid diet for the duration of the 3 month treatment (48% carbohydrate, 14.3% protein, and 4% fat; 2.9 kcal/g energy density). The food provided was the 2014 Teklad global 14% protein rodent maintenance diet (Envigo, Indianapolis, IN, USA). Daily food and fluid intake in all groups were carefully measured for a 3 month period, and no differences were observed between them (Figure 5). No health problems were shown. Based on previous studies conducted with the same treatment, we knew that the blood ethanol levels would be comparable in the ethanol-treated WT and TLR4-KO mice (125 ± 20 mg/dL) [46]. Finally, body weight gain at the end of the 3 month period was similar in all groups (Figure 1). Fecal samples were collected from all experimental groups (F-WT, F-WT-Et, F-KO, F-KO-Et, M-WT, M-WT-Et, M-KO, and M-KO-Et) at the end of the ethanol treatment (5 month-old animals). Stool pellets were collected fresh and individualized, immediately frozen in liquid nitrogen, and stored at −80 °C until further analysis. The experimental design is shown in Figure 5.

### 4.2. Bacterial 16S rRNA Library Construction and Sequencing

According to the manufacturer’s protocol, DNA was extracted using a QIAamp Fast DNA Stool Mini Kit (Qiagen, Hilden, Germany) with the following modifications: the stool and tissue samples were homogenized in 100 µL of InhibitEx buffer for 5 min at 95 °C. To elute the DNA, 100 µL of Buffer ATE was applied. The quality of the isolated DNA control was assessed using a spectrophotometer (NanoDrop 2000; Thermo Fisher Scientific, Waltham, MA, USA). The extracted bacterial DNA was stored at -20 °C until further use in the PCR amplification and sequencing.

The prokaryotic 16S ribosomal RNA gene (16S rRNA) libraries were generated from the extracted DNA using amplicon PCR for the variable V3-V4 region and following the Illumina 16S Sample Preparation Guide (San Diego, CA, USA). Initially, the region of interest was amplified using the specific primers containing overhang adapters (Table S2). Unbound primers, primer-dimer fragments, and other contaminants were removed with AMPure XP beads (Illumina) according to the above guide. A second PCR was performed using Nextera XT Index 1 Primers (N7XX) and 2 Primers (S5XX) (Illumina). Supplementary Table S2 shows the employed index.

The final quality and sizes of the end products were assessed by a TapeStation High Sensitivity DNA 1000 assay (Agilent Technologies, Santa Clara, CA, USA). The concentrations were evaluated using a Qubit^®^ dsDNA BR Assay kit (Thermo Fisher Scientific). Using the amplicon size and Qubit concentrations, the samples were normalized to 10 nM. A pool of amplicons at 10 nM was created by adding 2.5 µL of each normalized amplicon to a single pool. The pool was requantified by a TapeStation High Sensitivity DNA 1000 assay (Agilent Technologies) and Qubit. Sequencing was performed on the Illumina MiSeq platform (Illumina) following a 250 bp paired-end protocol according to the manufacturer’s specifications, with the addition of 25% PhiX. The library concentration was optimized to 8 pM. The experimental design is shown in Figure 5.

### 4.3. Bioinformatic Analysis

We processed raw sequence reads and generated the final ASV table following the standard DADA2 pipeline (https://benjjneb.github.io/dada2/tutorial.html) (accessed on 14 June 2023) [47].

#### 4.3.1. Data Quality Control and Filtering

First, we assessed sequencing quality using FastQC (v0.11.9) and MultiQC (v1.8) for a comprehensive view of read quality across the samples. We applied identical filters for both the male and female reads using the DADA2′s filterAndTrim function, which included the following: trimming the first 10 nucleotides from both the forward and reverse reads, setting a maximum of zero N nucleotides, removing the reads with more than the specified maximum expected errors (maxEE), limiting the forward reads to 2 expected errors and reverse reads to 5, and excluding the reads with a minimum length of under 75 nucleotides. The raw FASTQ files are publicly available at https://zenodo.org/records/14041366 (accessed on 5 November 2024).

#### 4.3.2. ASV Table Generation and Taxonomic Assignment

We conducted the subsequent steps (learning error rates, sample inference, merging paired reads, constructing the sequence table, and removing chimeras) using the default settings. Taxonomic assignment was performed against the Silva database (version 138.1).

#### 4.3.3. Community Structure and Diversity Analyses

Alpha diversity (species richness) was evaluated using the Chao1, Shannon, and Simpson indices to measure richness and evenness within samples. Beta diversity was assessed through Bray–Curtis-based non-metric multidimensional scaling (NMDS) to visualize the group differences, with significance determined by PERMANOVA to assess clustering between the groups. Both alpha and beta diversity were calculated with the phyloseq package, while PERMANOVA was conducted with the adonis2 function from the vegan package.

#### 4.3.4. Differential Abundance Analysis

We performed the differential abundance testing with the DESeq2 package, which adjusts for differences in library size and sequencing depth by modeling count data. To further control for library size variability, we used the poscount argument in DESeq2, followed by the Benjamini–Hochberg method for multiple comparison correction (FDR adjustment). Only the taxa with adjusted *p*-values of <0.05 were considered significantly different.

## 5. Conclusions

Our results demonstrated that chronic alcohol consumption induced sex differences in the microbiota profiles of WT male and female mice, with a higher abundance of bacteria with immunogenic capacity in the male mice. In addition, the deficiencies in the TLR4 receptor were capable of altering the microbiota contents in the ethanol-treated TLR4-KO mice of both sexes, leading to the higher enrichment of bacterial species associated with anti-inflammatory properties and the protection of the intestinal barrier, promoting a phenotype more resistant to the effects of ethanol. These findings can open up new avenues to understand the relationship between the involvement of gut microbiota profiles in the inflammation of the digestive system induced by chronic alcohol consumption. Therefore, a better understanding of the relationship between the gut microbiota and TLR4 activation should bring novel therapeutic measures to improve the balancing of the innate immune response and intestinal epithelial barrier function.

## Figures and Tables

**Table 1:** Summary of the differential abundance analysis. The over-represented taxa indicate significantly more abundant taxa (adjusted *p*-value of <0.05) in the first group compared to the second. The under-represented taxa indicate significantly less abundant taxa in the first group compared to the second. The non-differentially represented taxa indicate no significant differences in abundance (adjusted *p*-value of >0.05) between the groups.

	Differentially Abundant Taxa		
Comparison	Under-Represented	Non Diff-Represented	Over-Represented
F-WT-Et vs. M-WT-Et	25	625	13
F-WT-Et vs. F-WT	15	601	14
M-WT-Et vs. M-WT	14	654	11
F-WT-Et vs. F-KO-Et	27	581	22
M-WT-Et vs. M-KO-Et	15	642	37

**Table 2:** Differential expression in the bacteria phylum in the following comparisons: F-WT-Et vs. M-WT-Et, F-WT-Et vs. F-WT, and M-WT-Et vs. M-WT. The reads of each ASV were assigned to their respective phylum, and the differential expression was conducted using the DESeq2 package. Only the phyla with the lowest adjusted *p*-values (the most significant *p*-values for each comparison) are shown.

	ASV	BaseMean	Log2 Fold Change	*p*-Value	*p*-Adj	Phylum
F-WT-Et vs. M-WT-Et	ASV14	158.7571	−22.7539	2.4563 × 10^−54^	2.3532 × 10^−51^	*Bacteroidota*
	ASV19	130.0756	−22.7215	5.4090 × 10^−48^	2.5909 × 10^−45^	*Bacteroidota*
	ASV9	211.5929	−23.6201	6.5203 × 10^−34^	2.0821 × 10^−31^	*Firmicutes*
F-WT-Et vs. F-WT	ASV9	211.5929	−23.2442	6.90612 × 10^−33^	5.3039 × 10^−30^	*Firmicutes*
	ASV93	44.7947	23.34074	7.3835 × 10^−32^	2.8353 × 10^−29^	*Firmicutes*
	ASV34	91.2388	−24.3693	1.3663 × 10^−28^	3.4978 × 10^−26^	*Firmicutes*
M-WT-Et vs. M-WT	ASV14	158.7571	23.3235	6.99656 × 10^−68^	6.0380 × 10^−65^	*Bacteroidota*
	ASV19	130.0756	23.6099	3.51042 × 10^−61^	1.5148 × 10^−58^	*Bacteroidota*
	ASV85	53.5526	23.3412	2.8560 × 10^−41^	8.2157 × 10^−39^	*Bacteroidota*

**Table 3:** Differential expression in the bacterial phylum in the following comparisons: F-WT-Et vs. F-KO-Et and M-WT-Et vs. M-KO-Et. The reads of each ASV were assigned to their respective phylum, and the differential expression was conducted using the DESeq2 package. Only the phyla with the lowest adjusted *p*-values (the most significant *p*-values for each comparison) are shown.

	ASV	BaseMean	Log2 Fold Change	*p*-Value	*p*-Adj	Phylum
F-WT-Et vs. F-KO-Et	ASV14	158.7571	−26.1294	2.2666 × 10^−71^	2.2144 × 10^−68^	*Bacteroidota*
	ASV19	130.0756	−25.7657	2.0256 × 10^−61^	9.8949 × 10^−59^	*Bacteroidota*
	ASV213	15.5657	46.8328	3.6968 × 10^−54^	1.2039 × 10^−51^	*Firmicutes*
M-WT-Et vs. M-KO-Et	ASV175	21.2626	30.5586	2.2029 × 10^−38^	1.6103 × 10^−35^	*Bacteroidota*
	ASV335	8.7071	48.2812	6.3828 × 10^−34^	2.3329 × 10^−31^	*Firmicutes*
	ASV201	17.4193	47.6912	1.1312 × 10^−32^	2.7565 × 10^−30^	*Firmicutes*

## Data Availability

The data presented in this publication were deposited in the SRA public repository and are accessible through the following accession ID: PRJNA780249 (https://zenodo.org/records/14041366) (accessed on 5 November 2024).

## Supplementary Materials

The following supporting information can be downloaded at: https://www.mdpi.com/article/10.3390/ijms252312534/s1.
