# Supplementary material for: Metagenomics Reveals Sex-Based Differences in Murine Fecal Microbiota Profiles Induced by Chronic Alcohol Consumption
Source: Int J Mol Sci. 2024 Nov 22;25(23):12534. doi: 10.3390/ijms252312534 (PMC11641112; doi:10.3390/ijms252312534)
Supplement: Supplementary file 1 [file ijms-25-12534-s001.zip › ijms-3279284-supplementary.pdf]

SUPPLEMENTARY MATERIAL

TABLES

Table S1. Samples used in the study with their total reads and final viable readings.

Group	Sample	Index S	Sequence S	Index N	Sequence N	Total reads	Final reads %	Final reads
**F-WT**	F-WT-1	S502	CTCTCTAT	N703	AGGCAGAA	48713	84.1 %	40968
	F-WT-2	S502	CTCTCTAT	N704	TCCTGAGC	46719	84.2 %	39337
	F-WT-3	S504	AGAGTAGA	N703	AGGCAGAA	32173	84.2 %	27090
	F-WT-4	S504	AGAGTAGA	N704	TCCTGAGC	37896	83.4 %	31605
	F-WT-5	S504	AGAGTAGA	N706	TAGGCATG	5321	91.4 %	4863
	F-WT-6	S517	GCGTAAGA	N701	TAAGGCGA	7213	91.9 %	6629
**M-WT**	M-WT-1	S503	TATCCTCT	N702	CGTACTAG	61725	64.2 %	39627
	M-WT-2	S503	TATCCTCT	N701	TAAGGCGA	51591	68.2 %	35185
	M-WT-3	S503	TATCCTCT	N703	AGGCAGAA	31108	62.5 %	19443
	M-WT-4	S503	TATCCTCT	N704	TCCTGAGC	23280	58.1 %	13526
	M-WT-5	S503	TATCCTCT	N706	TAGGCATG	24680	62.6 %	15450
	M-WT-6	S504	AGAGTAGA	N701	TAAGGCGA	23960	62.7 %	15023
	M-WT-7	S504	AGAGTAGA	N702	CGTACTAG	7144	62.1 %	4436
	M-WT-8	S504	AGAGTAGA	N701	TAAGGCGA	18253	65.9 %	12029
**F-WT-Et**	F-WT-Et-1	S504	AGAGTAGA	N701	TAAGGCGA	44728	84.0 %	37572
	F-WT-Et-2	S504	AGAGTAGA	N702	CGTACTAG	52314	83.6 %	43735
	F-WT-Et-3	S504	AGAGTAGA	N703	AGGCAGAA	9709	91.2 %	8855
	F-WT-Et-4	S503	TATCCTCT	N705	GGACTCCT	65638	89.2 %	58549
	F-WT-Et-5	S502	CTCTCTAT	N701	TAAGGCGA	32568	81.5 %	26543
	F-WT-Et-6	S502	CTCTCTAT	N702	CGTACTAG	23445	87.2 %	20444
**M-WT-Et**	M-WT-Et-1	S503	TATCCTCT	N705	GGACTCCT	20518	65.7 %	13480
	M-WT-Et-2	S503	TATCCTCT	N703	AGGCAGAA	23998	56.9 %	13655
	M-WT-Et-3	S503	TATCCTCT	N704	TCCTGAGC	21630	63.1 %	13649
	M-WT-Et-4	S503	TATCCTCT	N706	TAGGCATG	18971	71.3 %	13526
	M-WT-Et-5	S502	CTCTCTAT	N704	TCCTGAGC	31558	74.4 %	23479
	M-WT-Et-6	S502	CTCTCTAT	N703	AGGCAGAA	21989	57.4 %	12622
**F-KO**	F-KO-1	S502	CTCTCTAT	N701	TAAGGCGA	45987	84.1 %	38675
	F-KO-2	S502	CTCTCTAT	N702	CGTACTAG	48941	83.6 %	40915
	F-KO-3	S504	AGAGTAGA	N702	CGTACTAG	29598	89.2 %	26401
	F-KO-4	S504	AGAGTAGA	N703	AGGCAGAA	7188	91.5 %	6577
	F-KO-5	S503	TATCCTCT	N705	GGACTCCT	60956	84.8 %	5169
	F-KO-6	S503	TATCCTCT	N706	TAGGCATG	59861	84.6 %	50642
**M-KO**	M-KO-1	S502	CTCTCTAT	N706	TAGGCATG	89854	70.9 %	63706
	M-KO-2	S502	CTCTCTAT	N705	GGACTCCT	99983	71.3 %	71288
	M-KO-3	S517	GCGTAAGA	N701	TAAGGCGA	17907	60.6 %	10852
	M-KO-4	S517	GCGTAAGA	N702	CGTACTAG	27921	63.8 %	17814
	M-KO-5	S517	GCGTAAGA	N703	AGGCAGAA	48240	46.3 %	22335
	M-KO-6	S517	GCGTAAGA	N704	TCCTGAGC	18126	52.2 %	9462
	M-KO-7	S517	GCGTAAGA	N705	GGACTCCT	18686	56.2 %	10502
	M-KO-8	S517	GCGTAAGA	N706	TAGGCATG	18654	58.6 %	10931
**F-KO-Et**	F-KO-Et-1	S503	TATCCTCT	N703	AGGCAGAA	58173	84.4 %	49098
	F-KO-Et-2	S503	TATCCTCT	N704	TCCTGAGC	49473	84.4 %	41755
	F-KO-Et-3	S504	AGAGTAGA	N706	TAGGCATG	32243	91.4 %	29470
	F-KO-Et-4	S517	GCGTAAGA	N701	TAAGGCGA	30386	92.8 %	28198
	F-KO-Et-5	S517	GCGTAAGA	N704	TCCTGAGC	45582	93.2 %	42482
	F-KO-Et-6	S517	GCGTAAGA	N705	GGACTCCT	51986	89.6 %	46579
**M-KO-Et**	M-KO-Et-1	S504	AGAGTAGA	N706	TAGGCATG	16115	61.23 %	9862
	M-KO-Et-2	S504	AGAGTAGA	N703	AGGCAGAA	16102	57.15 %	9210

M-KO-Et-3	S504	AGAGTAGA	N704	TCCTGAGC	13019	61.70 %	8033
M-KO-Et-4	S504	AGAGTAGA	N705	GGACTCCT	13101	62.35 %	8175
M-KO-Et-5	S502	CTCTCTAT	N701	TAAGGCGA	16241	70.75 %	11499
M-KO-Et-6	S502	CTCTCTAT	N702	CGTACTAG	48185	61.10 %	29441

Table S2. Primer sequences for the targeted mouse genes (qPCR assay).

Gene	Primer sequences (5' to 3')
**16S rRNA V3–V4 region**	**F:** TCGTCGGCAGCGTCAGATGTGTATA AGAGACAGCCTACGGGNGGCWGCAG<br>**R:** GTCTCGTGGGCTCGGAGATGTGTATAA GAGACAGGACTACHVGGGTATCTAATCC
